# Transactions of Chicago Society of Physicians and Surgeons

**Published:** 1873-07-01

**Authors:** P. S. Hayes


TRANSACTIONS OF THE CHICAGO SOCIETY OF PHYSICIANS AND SURGEONS.—REPORTED BY P. S. HAYES, M.D.—*Meeting June 9th*, 1873. Dr. Walter Hay read, by request, a paper on the Therapeutic Action of Alcohol. He first gave an outline of its effects on circulation, respiration, and enervation. All of the phenomena thus produced may be attributed to paralysis of the vasso motor nerves, the inhibitory fibres of the pneumogastric, and a quasi-paralysis of the nerves and the hemispheres. Enervation finally reaches its lowest ebb, and when the person is fully under the influence of alcohol, vegetative life alone exerts its way. Paralysis exists in every phase of acute alcoholism.

After the reading of the paper, a general discussion ensued, in which Dr. Hay explained how it was that alcohol acted by overcoming nerve action in certain specified cases, where its use had been found to be advantageous.

Dr. W. C. Lyman reported four cases which had occurred in his practice. The first was a case of spurious pregnancy; the second was a case of malformation of the external genitalia, in a woman; the third a fracture of the cranium, with subsequent abscess and loss of brain substance, followed by recovery; the fourth was the formation of enormous scibalous masses, and their retention for a time in pouches formed in the colon. The cause was attributed to indigestion and constipation. The evacuation of these masses, which occurred at different periods, produced peritonitis, while the voiding of the last was followed by hæmorrhage and death.

The meeting then adjourned.

THE THERAPEUTIC VALUE OF GELSEMINUM.—Dr. Philip C. Williams, Baltimore, Maryland (*Baltimore Medical Journal*), in an extended paper on the value of "Gelseminum," believes that it will cure all pure, simple neuralgias of the cerebral system with promptness and efficiency; that it will relieve cerebral congestion; that it controls maniacal excitement, and a variety of conditions resulting from derangements of the central nervous system. For these and analogous disorders he recommends gelseminum as a most powerful and efficient remedy.—*Southern Medical Record*.

